# Immune- and Stromal-Associated Tissue-Context Profiles in Pretreatment Triple-Negative Breast Cancer: An Exploratory Institutional Cohort Study

**DOI:** 10.3390/diseases14100367

**Published:** 2026-10-03

**Authors:** Valeria Zuccalà, Nicoletta Staropoli, Caterina Labanca, Gabriele Ricciardi, Pietro Tralongo, Vincenzo Fiorentino, Elisabetta Procopio, Maria Renne, Antonio Ieni, Maurizio Martini, Pierfrancesco Tassone, Pierosandro Tagliaferri

**Affiliations:** 1 Division of Pathology, Department of Human Pathology of Adults and Developmental Age “Gaetano Barresi”, University of Messina, 98125 Messina, Italymaurizio.martini@unime.it (M.M.); 2 Medical Oncology Unit, “R. Dulbecco” Teaching Hospital (Mater Domini Facility), Magna Græcia University and Cancer Center, Campus Salvatore Venuta, 88100 Catanzaro, Italy; 3 Department of Experimental and Clinical Medicine, Magna Græcia University, Campus Salvatore Venuta, 88100 Catanzaro, Italy; 4 Department of Biomedical, Dental, Morphological and Functional Imaging Sciences, University of Messina, 98125 Messina, Italy; 5 Pathology Unit, “Renato Dulbecco” Hospital, 88100 Catanzaro, Italy; 6 Surgery Unit, “R. Dulbecco” Teaching Hospital (Mater Domini Facility), Magna Græcia University and Cancer Center, Campus Salvatore Venuta, 88100 Catanzaro, Italy

**Keywords:** triple-negative breast cancer, tissue-context profile, stromal tumor-infiltrating lymphocytes, PD-L1, tumor-associated macrophages, mast cells, androgen receptor, germline *BRCA1*/*BRCA2*, hierarchical clustering

## Abstract

Background/Objectives: We examined combinations of immune- and stromal-associated features in pretreatment triple-negative breast cancer (TNBC) biopsies and explored their clinical and germline associations. Methods: This retrospective study included 24 women with nonmetastatic TNBC from a clinically selected *BRCA1*/*BRCA2*-tested cohort. Two pathologists, blinded to clinical and germline data, jointly assessed stromal tumor-infiltrating lymphocytes (sTILs), programmed death-ligand 1 (PD-L1), CD68/CD163-positive macrophages, and CD117/tryptase-positive mast cells. Profiles were assigned before unblinding. A second blinded joint sTIL assessment provided continuous estimates for subsequent hierarchical clustering; profile assignments remained unchanged. Results: Profiles I–III comprised seven, 13, and four tumors, respectively. Profile I combined higher sTILs and PD-L1 with a CD68-high/CD163-low pattern and rare mast cells. Profile II showed lower sTILs and PD-L1, a CD68-low/CD163-high pattern, and dense mast-cell hotspots; Profile III had minimal sTILs, low macrophage counts, and no mast cells detected in evaluated fields. Clustering reproduced these assignments. All four patients with pathogenic/likely pathogenic germline *BRCA1*/*BRCA2* variants were in Profile I (adjusted *p* = 0.030); omitting one positive case at a time increased the adjusted *p* value to 0.068. Seven of 16 neoadjuvant-treated patients achieved pathologic complete response. Five posttreatment recurrences and seven deaths were recorded; exploratory comparisons of pathologic complete response and overall survival were inconclusive. Conclusions: These profiles describe different combinations of lymphocytic infiltration, PD-L1 expression, macrophage counts, and mast-cell hotspots. The profiles and the exploratory *BRCA1*/*BRCA2* finding require validation in larger, independent cohorts before clinical use.

## 1. Introduction

Breast cancer remains the most commonly diagnosed malignancy among women worldwide. According to the International Agency for Research on Cancer (IARC)/World Health Organization (WHO) GLOBOCAN 2024 estimates, breast cancer accounted for approximately 2.43 million new cases and 693,660 deaths worldwide in both sexes combined, with an estimated 5-year prevalence of approximately 8.16 million people [1]. Separate IARC projections indicate that, if current rates persist, female breast cancer could reach approximately 3.2 million new cases and 1.1 million deaths annually by 2050 [2]. Contemporary cancer care increasingly combines established local and cytotoxic treatments with molecularly targeted and immunologic approaches [3]. Triple-negative breast cancer (TNBC) is defined by estrogen receptor (ER) and progesterone receptor (PR) expression below 1% together with the absence of human epidermal growth factor receptor 2 (HER2) overexpression or *ERBB2* amplification, according to the applicable American Society of Clinical Oncology/College of American Pathologists (ASCO/CAP) criteria [4,5,6]. Within conventionally HER2-negative breast cancer, recognition of HER2-low and ultra-low expression further illustrates the heterogeneity that can exist within clinically defined receptor categories [7]. TNBC itself comprises tumors with marked genomic, transcriptomic, morphological, and immunological heterogeneity [8,9]. Spatial profiling has shown tissue architectures ranging from inflamed to immune-excluded, compartmentalized, and immune-poor states [10,11,12], underscoring the biological and therapeutic relevance of the local tumor context [13].

Pretreatment core biopsies provide an opportunity to examine the local tissue context before systemic therapy, although they necessarily sample only a portion of a spatially heterogeneous lesion. Stromal tumor-infiltrating lymphocytes (sTILs), assessed according to the International TILs Working Group recommendations [14,15], have established prognostic relevance at the population level in early TNBC [16]. Programmed death-ligand 1 (PD-L1) expression can accompany lymphocyte-rich tumors and adaptive immune resistance, but neither sTILs nor PD-L1 alone captures the composition or functional state of the broader immune and stromal compartments [17,18].

Tumor-associated macrophages (TAMs) are another component of the local tissue environment. CD68 is a broad macrophage marker, whereas CD163 is associated with an immunoregulatory phenotype; neither marker alone is sufficient to assign an M1 or M2 functional state [19,20,21]. The CD163/CD68 ratio can describe relative TAM composition, although its cutoffs are method- and cohort-dependent [20]. CD117 is expressed by mast cells but is not lineage-specific, making same-cell assessment of mast-cell tryptase useful for lineage confirmation. In breast cancer, tryptase-positive mast cells and CD117/tryptase-expressing mast cells have been associated with microvascular and angiogenic features [22,23], while c-KIT signaling has also been implicated in TNBC cell migration [24].

Tumor-cell phenotype and hereditary predisposition provide additional context for interpreting these tissue findings. Androgen receptor (AR) expression describes an accompanying epithelial phenotype, and androgen-related differentiation has been investigated in relation to immune features and treatment response in TNBC [25,26]. *BRCA1* and *BRCA2* encode proteins involved in homologous-recombination repair of DNA double-strand breaks. Germline loss-of-function variants confer hereditary breast cancer predisposition, with *BRCA1*-associated cancers particularly enriched for the triple-negative phenotype [27,28,29]. Germline status also informs risk management and treatment, including the use of poly (ADP-ribose) polymerase (PARP) inhibitors [27,29]. Examining these tissue patterns alongside germline status may therefore help characterize a clinically important subset of TNBC.

This exploratory study aimed to describe immune- and stromal-associated profiles in pretreatment TNBC core biopsies and examine their relationships with clinicopathological features and germline *BRCA1*/*BRCA2* status. We also used quantitative tissue measurements to assess whether clustering reproduced the profiles identified by the pathologists. Body mass index, treatment response, and follow-up outcomes were examined as additional exploratory variables.

## 2. Materials and Methods

### 2.1. Study Design and Cohort

This retrospective, observational, single-institution cohort study included women diagnosed with invasive TNBC between 2018 and 2021 at the Pathology and Medical Oncology Units of Magna Græcia University and the R. Dulbecco Teaching Hospital, Catanzaro, Italy. Eligibility required adult age, nonmetastatic disease, sufficient pretreatment formalin-fixed, paraffin-embedded (FFPE) core biopsy material for histological and immunohistochemical assessment, and a completed final clinical germline *BRCA1*/*BRCA2* report. Each patient contributed a single diagnostic episode and one pretreatment index biopsy. An institutional query identified 31 potentially eligible women; seven were excluded because residual pretreatment tissue was insufficient (*n* = 3), the required immunohistochemical panel was incomplete (*n* = 2), systemic therapy preceded the index biopsy (*n* = 1), or the available pretreatment diagnostic specimen was not a core biopsy (*n* = 1). The final study cohort therefore comprised 24 patients.

TNBC status was verified on the pretreatment index core biopsy: ER and PR expression had to be <1% of viable invasive-tumor nuclei, with HER2 negativity established according to the applicable ASCO/CAP criteria [4,5]. HER2-negative cases were immunohistochemistry (IHC) 0 or 1+ or IHC 2+ with a negative integrated IHC/dual-probe in situ hybridization (ISH) result under the 2018 algorithm. Missing or unresolved receptor results precluded inclusion. Diagnostic reports were checked using the 2020 ER/PR and 2018 HER2 definitions, without changing cohort composition. Ready-to-use ER SP1, PR 1E2, and HER2 4B5 assays (Roche/Ventana Medical Systems, Tucson, AZ, USA) were performed on BenchMark XT with ultraView Universal DAB detection (both Roche/Ventana Medical Systems, Tucson, AZ, USA). External controls were included, and benign mammary epithelium provided an internal ER/PR control when present. HER2 IHC 2+ cases underwent INFORM HER2 Dual ISH (Roche/Ventana Medical Systems, Tucson, AZ, USA) on the same platform, with silver detection for *ERBB2* and red detection for chromosome 17; interpretation included second-observer recount and adjudication when required by the 2018 algorithm.

During 2018–2021, 74 women were diagnosed with nonmetastatic TNBC at the institution; 31 (41.9%) completed clinical germline *BRCA1*/*BRCA2* testing. Testing was offered through routine clinical-genetics assessment, considering age at diagnosis, bilateral or multiple primary breast cancers, personal history of ovarian or pancreatic cancer, and family history of breast, ovarian, pancreatic, or prostate cancer. Referral criteria did not materially change during the study period. Of these 31 tested patients, the 24 who met the tissue eligibility criteria were included. Detailed family history data were not extracted for analysis, and ancestry information was unavailable.

Patient identifiers were replaced with study codes. Reporting followed the STROBE recommendations for cohort studies [30].

### 2.2. Pretreatment Clinical Staging and Clinicopathological Variables

Pretreatment clinical anatomic stage was assigned according to the eighth edition of the American Joint Committee on Cancer (AJCC) staging system [31]. Clinical T and N categories were based on examination, mammography, breast and axillary ultrasonography, magnetic resonance imaging when indicated, and needle sampling of suspicious lymph nodes [32]. Systemic staging was used as appropriate to document cM0 disease. All staging variables referred to the assessment before systemic therapy, including for patients subsequently treated with neoadjuvant chemotherapy [33].

Age, pretreatment clinical stage and nodal status, the original histological diagnosis, Ki-67, and germline *BRCA1*/*BRCA2* status were abstracted from institutional records. Lymphovascular invasion (LVI) and perineural invasion (PNI) were assessed jointly by Valeria Zuccalà (V.Z.) and Antonio Ieni (A.I.) on hematoxylin and eosin (H & E) sections of the pretreatment index biopsy, while clinical and germline information remained blinded. LVI required unequivocal tumor emboli in endothelial-lined spaces, excluding retraction artifact. PNI required unequivocal neoplastic cells surrounding and encircling a nerve. Both findings were recorded at the initial study assessment and were treated as secondary comparisons because of their limited representation in core biopsies.

Histological grade was not an eligibility criterion. All 24 tumors were Nottingham histological grade 3, so grade was treated as a uniform cohort characteristic and was not included in inferential analyses.

Pretreatment height and weight, treatment sequence, pathologic complete response (pCR), recurrence, death, and last clinical contact were abstracted from the clinical files and cross-checked before analysis. Records were reviewed and updated through January 2026; individual follow-up ended at the documented death or last clinical contact, with the latest contact recorded on 27 December 2024. Body mass index (BMI) was calculated as weight in kilograms divided by height in meters squared, and obesity was defined as BMI ≥ 30 kg/m^2^. Neoadjuvant therapy was defined as systemic treatment initiated before surgery. All 16 neoadjuvant-treated patients received doxorubicin plus cyclophosphamide (AC) or epirubicin plus cyclophosphamide (EC), followed by paclitaxel or docetaxel. Final surgical pathology was available for all 24 patients. pCR, defined as ypT0/is ypN0, was evaluable in all 16 neoadjuvant-treated patients.

Overall survival (OS) was measured from the index biopsy to death from any cause or last known contact. Primary treatment was considered complete when all planned surgical, systemic, and radiotherapy components had been completed. Recurrence was defined as the first documented locoregional and/or distant recurrence after that completion date. Disease events occurring before treatment completion were recorded separately. Time from the index biopsy to first recurrence and the pattern of recurrence were summarized descriptively.

### 2.3. Histopathological Records, sTIL Assessment, and Tissue-Context Profile Recognition

Histological categories were taken from the original diagnostic reports and harmonized to WHO terminology [34], without retrospective histotype reassignment. During the initial study review, V.Z. and A.I. jointly assessed Nottingham grade [35], confirming the original grade in all 24 tumors.

V.Z. and A.I. assessed the pathological features jointly, recording consensus results rather than independent reader scores. sTILs were estimated as the percentage of tumor-associated stromal area occupied by mononuclear inflammatory cells, following the International TILs Working Group principles [14,15]. The whole evaluable invasive-tumor stroma across all core specimens was assessed, rather than selected hotspots. In situ carcinoma, normal breast structures, necrosis, crush artifact, hyalinized scar, and stroma without adjacent viable invasive tumor were excluded. Lymphocytes and plasma cells were included; granulocytes were excluded.

All H & E-based and immunohistochemical assessments and profile assignment were completed while both pathologists were blinded to age, pretreatment cT, cN and clinical stage, Ki-67, and germline *BRCA1*/*BRCA2* status.

During the initial joint slide review, V.Z. and A.I. considered sTIL levels, PD-L1 combined positive score (CPS), CD68/CD163-positive macrophage counts, and stromal mast-cell counts together for each case. They recognized three patterns and assigned all 24 tumors by consensus before clinical or germline data were disclosed. The profiles emerged from recurring tissue patterns, without a predefined classification rule or target number of groups. The numerical categories described below were applied afterward to summarize the marker distributions.

AR, LVI, PNI, the derived CD163/CD68 ratio, and histomorphology were recorded separately and did not determine profile assignment. Tryptase confirmed mast-cell identity and was not a separate grouping variable. Since the same markers informed profile recognition and description, their differences across profiles were treated descriptively, not as independent validation. Interobserver agreement could not be estimated from consensus scores.

At the initial review, sTILs were estimated as percentages, but exact case-level values were not retained for every tumor; the worksheet preserved point estimates, intervals, or upper bounds. After profile assignments had been finalized, V.Z. and A.I. performed a second joint review of the original H & E slides to obtain a numerical sTIL percentage for each of the 24 tumors. During this second review, they remained blinded to all clinical and germline information, as well as the assigned profiles and the original sTIL notation. The new estimates were read directly from the slides and did not change profile assignment. Only sTILs were reassessed. Subsequent quantitative analyses used these second-assessment percentages together with the original CPS and cell counts. The initial sTIL notation and the second-assessment percentages are reported separately. These visually estimated percentages were not considered measurements with decimal-level precision.

### 2.4. Immunohistochemistry, Tumor-Associated Macrophage Measurements, and Mast-Cell Assessment

Biopsies were placed in 10% neutral-buffered formalin within 1 h, fixed for 12–72 h, and processed to paraffin. The block with the greatest amount of viable invasive tumor and associated stroma was selected. Sections were cut onto charged slides, stored in the dark at 2–8 °C, and stained within 14 days. An adjacent H & E section confirmed retained tumor and stroma. Sections with tissue loss, folds, exhausted tumor, or inadequate controls were repeated; all 24 cases were evaluable.

PD-L1 was assessed with the complete 22C3 pharmDx assay (Agilent/Dako, Carpinteria, CA, USA) on Autostainer Link 48 (Agilent/Dako, Glostrup, Denmark), using 4–5 µm sections, low-pH EnVision FLEX retrieval in PT Link and the corresponding horseradish peroxidase (HRP)/DAB detection system (Agilent/Dako, Glostrup, Denmark). Manufacturer-specified cell-line, tissue, and reagent controls were included in each run. V.Z. and A.I. jointly reviewed the whole viable invasive-tumor area at a dual-head microscope; every case met the requirement of at least 100 viable invasive-tumor cells. CPS was calculated as the number of PD-L1-positive tumor cells, lymphocytes, and macrophages divided by the number of viable invasive-tumor cells, multiplied by 100 and capped at 100. Tumor-cell positivity required membranous staining; tumor-associated lymphocytes and macrophages could show membrane and/or cytoplasmic staining. Cytoplasmic-only tumor staining and unrelated inflammatory cells were excluded. CPS was also summarized as <10 or ≥10, without using this threshold alone to assign profiles. The threshold has a treatment selection role in advanced TNBC [17,36], whereas KEYNOTE-522 enrolled stage II–III patients irrespective of PD-L1 status [37]; it was descriptive in this nonmetastatic cohort.

Ki-67 was assessed on 4 µm index biopsy sections using ready-to-use clone 30-9 (Roche/Ventana Medical Systems, Tucson, AZ, USA), BenchMark XT, and ultraView Universal DAB detection. Tonsil and, when present, non-neoplastic proliferating elements served as external and internal controls. At least 500 viable invasive-tumor nuclei were evaluated across areas with higher and lower labeling in proportion to their extent, counting unequivocal nuclear staining as positive. The percentage recorded in the diagnostic report was used without a cutoff.

CD68 and CD163 were assessed on 4 µm sections using clones KP1 (Agilent/Dako, Glostrup, Denmark; M0814; 1:100) and 10D6 (Leica Biosystems, Newcastle upon Tyne, UK; NCL-L-CD163; 1:200), respectively, on BOND-III (Leica Biosystems, Melbourne, Australia) after heat-induced antigen retrieval, with BOND Polymer Refine Red detection (Leica Biosystems, Newcastle upon Tyne, UK; DS9390; alkaline phosphatase/Fast Red). Tonsil and placenta were the respective external controls; resident histiocytes provided internal controls. Positive TAMs required macrophage morphology with granular cytoplasmic CD68 staining or membranous and/or cytoplasmic CD163 staining. Epithelial and lymphocytic staining was excluded. The pathologists jointly counted positive cells in five matched, non-overlapping 400× stromal fields per stain, distributed across viable invasive-tumor stroma and matched on serial sections using anatomical landmarks. Substantial invasive epithelium, necrosis, artifact, in situ carcinoma, normal breast tissue, and extra tumoral lymphoid aggregates were avoided. All cases provided five suitable fields. An Olympus BX53 microscope (Olympus Corporation, Tokyo, Japan) with 10× eyepiece, field number 22, and 40× objective gave a gross field area of 0.238 mm^2^. Five-field counts were averaged without normalization to the stromal area within each field; the CD163/CD68 ratio was calculated from the paired means.

Same-section dual CD117/tryptase staining was performed on 5 µm FFPE sections using rabbit polyclonal CD117/c-KIT (Agilent/Dako, Glostrup, Denmark; A4502; 1:100) and mouse monoclonal tryptase AA1 (Agilent/Dako, Glostrup, Denmark; M7052; 1:100). After automated deparaffinization and 20 min of retrieval with BOND Epitope Retrieval Solution 2 (Leica Biosystems, Newcastle upon Tyne, UK), the antibody cocktail was applied for 30 min at room temperature on BOND-III. ChromoPlex 1 Dual Detection (Leica Biosystems, Newcastle upon Tyne, UK; DS9477) produced red alkaline-phosphatase labeling for CD117 and brown HRP/DAB labeling for tryptase, with hematoxylin counterstaining and red-chromogen-compatible mounting.

Normal skin served as the dual-positive control, and gastrointestinal stromal tumor as a CD117-positive/tryptase-negative control. Single-primary and no-primary controls checked for cross-detection and nonspecific staining. Runs were accepted only with the expected control reactions. The dual assay had been verified against single-marker stains on non-study FFPE breast specimens.

Countable mast cells were intact, nucleated stromal cells with compatible morphology and same-cell membranous and/or cytoplasmic CD117 staining plus granular cytoplasmic tryptase staining. Epithelial staining, CD117-positive/tryptase-negative cells, extracellular tryptase deposits, necrotic cells, and artifacts were excluded.

All viable specimens were surveyed at 100×, followed by joint counting in the five non-overlapping 400× tumor-associated stromal fields with the most dual-positive mast cells. When none were seen on the survey, five viable stromal fields were nevertheless examined at 400×. The same microscope and 0.238 mm^2^ field area were used as for TAMs. Counts were summed and divided by five to give each patient’s mean hotspot count. A zero count meant no mast cells detected in the evaluated fields, not absence throughout the tumor.

AR was assessed with clone AR441 (Agilent/Dako, Glostrup, Denmark; M3562; 1:50) on BenchMark XT with ultraView Universal DAB detection. Unequivocal nuclear staining in viable invasive-tumor cells was estimated jointly across the evaluable tumor. The results were described as negative (<1%) or positive (≥1%); this threshold did not establish the transcriptomic luminal androgen receptor subtype or treatment eligibility.

The CD68, CD163, and AR protocols had been optimized and analytically verified on non-study FFPE specimens with appropriate positive and negative controls, checking staining localization and signal-to-background performance.

For descriptive reporting, mean CD68 and CD163 counts were categorized as low (<10) or high (≥10 cells per 400× field); mean mast-cell counts as not detected (0), rare (>0 to 5), or dense (>5 cells per field); and CD163/CD68 ratios as CD68-predominant (<0.80), balanced (0.80–1.20), or CD163-predominant (>1.20). These categories were applied after profile assignment, without reference to germline or clinical variables. They are exploratory summaries of this cohort, not validated diagnostic thresholds. CD163 findings describe immunophenotypic composition, not M1/M2 polarization.

### 2.5. Germline BRCA1/BRCA2 Testing and Classification

All 24 patients had a finalized clinical germline *BRCA1*/*BRCA2* report. Variants were classified according to the American College of Medical Genetics and Genomics/Association for Molecular Pathology (ACMG/AMP) framework [38]. Patients were analyzed as having a documented pathogenic or likely pathogenic (P/LP) variant or no documented P/LP variant; variants of uncertain significance (VUS) were included in the latter category. Three patients carried a *BRCA1* P/LP variant and one a *BRCA2* P/LP variant, with no dual-gene carriers. Two other patients had a VUS, one in each gene. Comparable results for *PALB2*, *TP53*, *CHEK2*, *ATM*, or other susceptibility genes were not available across all 24 patients; no cohort-wide analysis of these genes was performed.

Peripheral-blood DNA was analyzed using an internally validated hybrid-capture next-generation sequencing (NGS) assay for *BRCA1* and *BRCA2*. Libraries were prepared using the SureSelectXT Reagent Kit with custom *BRCA1*/*BRCA2* capture probes (Agilent Technologies, Santa Clara, CA, USA) and sequenced on a NextSeq 550 platform (Illumina, San Diego, CA, USA). Coding exons and ±20-bp splice boundaries were covered at a mean sequencing depth > 500×; regions with coverage < 100× were completed by Sanger sequencing.

BCL files were demultiplexed and converted to FASTQ format using bcl2fastq2 v2.20.0 (Illumina), and reads were aligned to the GRCh37/hg19 reference genome using BWA-MEM v0.7.17. Germline single-nucleotide variants and small insertions/deletions were called using GATK v3.8 HaplotypeCaller and annotated using ANNOVAR (2017Jul16 release). Copy-number variants were assessed from NGS read-depth data using DECoN v1.0.1 and confirmed by multiplex ligation-dependent probe amplification (MLPA) using SALSA MLPA Probemix P002 *BRCA1* and P045 *BRCA2*/*CHEK2* with the SALSA MLPA Reagent Kit (MRC Holland, Amsterdam, The Netherlands); MLPA data were analyzed using Coffalyser.Net v140721.1958. Clinically reportable single-nucleotide variants and small insertions/deletions, as well as regions requiring coverage completion, were analyzed by Sanger sequencing using BigDye Terminator v1.1 chemistry on a SeqStudio Genetic Analyzer (Applied Biosystems/Thermo Fisher Scientific, Waltham, MA, USA). Variant annotation used transcripts NM_007294.4 for *BRCA1* and NM_000059.4 for *BRCA2*, and classification followed ACMG/AMP criteria supplemented by Evidence-based Network for the Interpretation of Germline Mutant Alleles (ENIGMA) guidance when applicable.

Three patients carried a *BRCA1* P/LP variant—NM_007294.4:c.4035del, p.(Glu1346LysfsTer20); NM_007294.4:c.5503C>T, p.(Arg1835Ter); and NM_007294.4:c.135-1G>T, p.?—and one carried a *BRCA2* P/LP variant, NM_000059.4:c.5946del, p.(Ser1982ArgfsTer22).

### 2.6. Ethics Approval and Consent to Participate

The study was conducted in accordance with the Declaration of Helsinki and was approved by the Ethics Committee of Regione Calabria, Sezione Area Centro (approval code: prot. no. 2012.73). All participants had provided written informed consent for the use of biological material and related clinical information for research purposes. Clinical germline *BRCA1*/*BRCA2* testing was performed as part of routine care under the applicable clinical consent procedures. No additional patient procedures were undertaken for this study.

### 2.7. Statistical Analysis

Analyses were performed in R 4.6.1 (R Foundation for Statistical Computing, Vienna, Austria). Categorical data were summarized as counts and percentages; continuous data were summarized as median, first and third quartiles (Q1–Q3) and range, with mean and standard deviation also given for age. Five exploratory comparisons were selected before clinical and germline unblinding: *BRCA1*/*BRCA2* status, clinical stage, cN status, age, and Ki-67. LVI and PNI were designated as separate secondary comparisons before their distributions across profiles were examined and before unblinding.

Categorical comparisons used two-sided Fisher–Freeman–Halton exact tests; age and Ki-67 used permutation Kruskal–Wallis tests (9,999,999 permutations). Effect sizes were Cramér’s V for categorical variables and eta-squared based on the Kruskal–Wallis statistic (η^2^_H_) for continuous variables. The exact Clopper–Pearson 95% confidence intervals were reported for germline P/LP frequencies and profile-specific proportions of LVI, PNI, cN-positive disease, stage III, pCR, death, and recurrence. η^2^_H_ was calculated as max [0, (H − k + 1)/(N − k)], with H denoting the tie-corrected Kruskal–Wallis statistic, k the number of groups, and *N* the number of patients.

The five main comparisons and their multiplicity adjustment were repeated four times, each time omitting a different P/LP-positive patient. These sensitivity analyses contained 23 patients; the main analyses retained all 24.

The Benjamini–Hochberg adjustment was applied to unrounded *p* values across the five main comparisons and, separately, across LVI and PNI. cN was analyzed as cN0 versus cN1–3; because cN contributes to stage, these were related comparisons. cT was descriptive. The sample comprised all eligible tested patients in the study period, without a prior sample-size calculation. Data for the comparisons selected before unblinding were complete, with no imputation. Multivariable models were not fitted.

The post hoc Ward hierarchical clustering used five quantitative inputs: the second-assessment sTIL percentage, original PD-L1 CPS, and original mean CD68-positive, CD163-positive, and dual-positive mast-cell counts. Clinical data and pathology profile labels were excluded from the input. Each variable underwent ln (1 + x) transformation and standardization to zero mean and unit variance. With the Euclidean distance, solutions of two to five clusters were compared using the mean silhouette, a measure of within-cluster similarity relative to other clusters. Agreement with the previously assigned profiles was then measured by the adjusted Rand index (ARI), where 1 indicates identical assignments.

Two sensitivity analyses repeated this procedure after rounding the second-assessment sTIL percentages to the nearest five percentage points and after omitting sTILs. These analyses examined whether the numerical grouping depended on the precision or inclusion of the second sTIL assessment; neither constituted independent validation.

BMI was compared using the Kruskal–Wallis test; obesity and pCR in neoadjuvant-treated patients used exact tests. These additional comparisons were exploratory and unadjusted for multiple testing. OS was compared by global log-rank test. Median follow-up was estimated by reverse Kaplan–Meier, treating deaths as censored observations in the follow-up distribution. Death and posttreatment recurrence counts were described as crude proportions of the whole cohort, not cumulative incidence estimates. Recurrence was also summarized among patients who completed primary treatment. No competing-risk or multivariable recurrence model was fitted.

## 3. Results

### 3.1. Cohort Characteristics

The cohort comprised 24 women with a mean age at diagnosis of 58.1 years (SD, 12.7) and a median age of 61 years (Q1–Q3, 48.3–67.0; range, 33–77). Twenty tumors (83.3%) were invasive breast carcinomas of no special type. The remaining four comprised one carcinoma with pleomorphic morphology, one carcinoma with apocrine differentiation, one metaplastic carcinoma with chondroid differentiation, and one metaplastic carcinoma with squamous differentiation.

Pretreatment clinical categories were cT1 in two patients, cT2 in 10, cT3 in six, and cT4 in six; cN0 in 11, cN1 in 11, cN2 in one, and cN3 in one. Thirteen patients had pretreatment clinical anatomic stage II disease, and 11 had pretreatment clinical anatomic stage III disease. All 24 tumors were Nottingham grade 3; grade 3 was an observed cohort characteristic and not an eligibility criterion.

LVI and PNI were identified in eight (33.3%) and five (20.8%) biopsies, respectively. Median Ki-67 was 60% (Q1–Q3, 40–72.5%; range, 10–90%). Stage, LVI, PNI, Ki-67, and finalized germline BRCA1/BRCA2 status were available for all 24 patients. Four carried a P/LP variant (16.7%; exact 95% CI, 4.7–37.4%): three in BRCA1 and one in BRCA2. Two additional VUS were retained in the no-documented P/LP category. BMI was available for all patients, with a median of 25.4 kg/m^2^ (Q1–Q3, 22.3–28.6; range, 19.7–36.3); five (20.8%) had obesity. Sixteen patients received neoadjuvant therapy, and seven achieved pCR. Median follow-up was 35 months (observed range, 5–60), with seven deaths and five posttreatment recurrences. Cohort characteristics and overall tissue findings are summarized in Table 1 and Table 2.

**Table 1:** Clinicopathological characteristics of the 24-case cohort.

Characteristic	Category or Statistic	Overall Cohort (*N* = 24)
Age, years	Mean ± SD	58.1 ± 12.7
	Median (Q1–Q3; range)	61 (48.3–67.0; 33–77)
Histological category	Invasive breast carcinoma of no special type	20 (83.3%)
	Carcinoma with pleomorphic morphology	1 (4.2%)
	Metaplastic carcinoma with chondroid differentiation	1 (4.2%)
	Carcinoma with apocrine differentiation	1 (4.2%)
	Metaplastic carcinoma with squamous differentiation	1 (4.2%)
Pretreatment clinical T category	cT1/cT2/cT3/cT4	2/10/6/6
Pretreatment clinical N category	cN0/cN1/cN2/cN3	11/11/1/1
Pretreatment clinical stage group	II/III	13 (54.2%)/11 (45.8%)
LVI on pretreatment biopsy	Absent/present	16 (66.7%)/8 (33.3%)
PNI on pretreatment biopsy	Absent/present	19 (79.2%)/5 (20.8%)
Histological grade	Nottingham grade 3	24 (100%)
Ki-67, %	Median (Q1–Q3; range)	60 (40–72.5; 10–90)
Germline *BRCA1*/*BRCA2* status	No documented P/LP variant	20 (83.3%)
	Documented P/LP variant	4 (16.7%; exact 95% CI, 4.7–37.4%)
	*BRCA1* P/LP variant	3 (12.5%)
	*BRCA2* P/LP variant	1 (4.2%)
	VUS, not counted as P/LP	2 (8.3%)
BMI, kg/m^2^	Median (Q1–Q3; range)	25.4 (22.3–28.6; 19.7–36.3)
Obesity	BMI ≥ 30 kg/m^2^	5 (20.8%)
Systemic treatment sequence	Neoadjuvant therapy	16 (66.7%)
pCR	Among neoadjuvant-treated patients	7/16 (43.8%)
Follow-up outcome	Deaths	7 (29.2%)
	Posttreatment recurrences	5 (20.8%)

Values are *n* (%) unless otherwise stated. Stage is pretreatment clinical anatomic stage; LVI and PNI refer to the index biopsy. The two VUS are included among the 20 patients without a documented P/LP variant. BMI was calculated from pretreatment height and weight. pCR (ypT0/is ypN0) was evaluable in all 16 neoadjuvant-treated patients. Death and recurrence percentages use the full cohort denominator; 23 patients completed primary treatment, whereas one had not completed it at last contact. Median follow-up by reverse Kaplan–Meier was 35 months (observed range, 5–60). BMI, body mass index; CI, confidence interval; LVI, lymphovascular invasion; P/LP, pathogenic or likely pathogenic; pCR, pathologic complete response; PNI, perineural invasion; Q1–Q3, first to third quartile; SD, standard deviation; VUS, variant of uncertain significance.

**Table 2:** Overall immune- and stromal-associated characteristics.

Parameter	Recorded Category	Overall Cohort (*N* = 24)
sTILs, initial assessment	<1%	4 (16.7%)
	1–10%	13 (54.2%)
	15–40%	7 (29.2%)
PD-L1 CPS	<10/≥10	17 (70.8%)/7 (29.2%)
CD68-positive TAMs, mean per 400× field	Low/high	17 (70.8%)/7 (29.2%)
CD163-positive TAMs, mean per 400× field	Low/high	11 (45.8%)/13 (54.2%)
CD163/CD68 TAM ratio category	CD68-predominant/balanced/CD163-predominant	7 (29.2%)/5 (20.8%)/12 (50.0%)
Stromal mast-cell hotspot category (CD117+/tryptase+)	Not detected/rare/dense	4 (16.7%)/7 (29.2%)/13 (54.2%)
AR	Negative/positive	18 (75.0%)/6 (25.0%)

The sTIL bands summarize the initial worksheet entries and are not prespecified assignment thresholds. No initial entry described a level >10% and <15%. The second-assessment sTIL percentages, summarized in Table 3, were used for clustering, not to reassign profiles. CD68/CD163 categories are low (<10) or high (≥10 cells per 400× field); mast-cell categories are not detected (0), rare (>0 to 5), or dense (>5 cells per field). Mast cells required same-cell CD117/tryptase staining and were counted in five fields under the hotspot protocol. “Not detected” refers only to those fields. Count and ratio categories are exploratory rather than diagnostic thresholds. AR, androgen receptor; CPS, combined positive score; PD-L1, programmed death-ligand 1; sTILs, stromal tumor-infiltrating lymphocytes; TAM, tumor-associated macrophage.

**Table 3:** Tissue and histomorphological features of the three tissue-context profiles.

Feature	Profile I (*n* = 7)	Profile II (*n* = 13)	Profile III (*n* = 4)
sTILs, initial worksheet notation	15–20% (1); 20% (1); 20–25% (3); 30% (1); 40% (1)	1–5% (3); 5% (1); 5–10% (2); 1–10% (7)	<1% (4)
sTILs, second assessment, %, median (Q1–Q3; range)	22.1 (20.0–27.1; 19.1–40.0)	4.9 (4.3–7.8; 1.0–9.2)	0.61 (0.51–0.69; 0.25–0.88)
PD-L1 CPS category	≥10 in 7/7	<10 in 13/13	<10 in 4/4
PD-L1 CPS, median (Q1–Q3; range)	22 (13.5–26; 11–40)	3 (0–7; 0–9)	7 (5.5–7; 1–7)
CD68/CD163 TAM mean-count categories	CD68 high; CD163 low	CD68 low; CD163 high	CD68 and CD163 low
CD68-positive TAM mean/field, median (Q1–Q3; range)	18.0 (15.5–18.6; 14.0–20.0)	7.6 (6.6–8.0; 6.0–9.4)	5.0 (4.0–6.1; 4.0–6.2)
CD163-positive TAM mean/field, median (Q1–Q3; range)	8.4 (8.2–9.0; 7.6–9.4)	13.0 (12.2–14.8; 11.0–16.0)	5.3 (4.3–6.1; 3.4–6.2)
Mast-cell hotspot count/field (CD117+/tryptase+), median (Q1–Q3; range)	2.4 (1.9–3.3; 1.2–4.2)	7.2 (6.0–10.4; 5.2–13.4)	0.0 (0.0–0.0; 0.0–0.0)
CD163/CD68 TAM ratio	0.50 (Q1–Q3, 0.44–0.57); CD68-predominant 7/7	1.85 (1.55–2.15); CD163-predominant 12/13; balanced 1/13	1.00 (0.94–1.06); balanced 4/4 at low mean counts
Mast-cell hotspot category (CD117+/tryptase+)	Rare in 7/7	Dense in 13/13	Not detected in 4/4
AR	Negative in 7/7	Positive in 6/13; negative in 7/13	Negative in 4/4
Histomorphology	No special type in 7/7	No special type in 12/13; apocrine differentiation in 1/13	Pleomorphic morphology (1); metaplastic chondroid (1); metaplastic squamous (1); no special type (1)

The first sTIL row reproduces the initial worksheet notation; numbers in parentheses are case counts, and the overlapping intervals are not mutually exclusive predefined bins. The second sTIL row summarizes the percentages used in the quantitative analyses. Decimal reporting of these visual estimates does not imply analytical measurement precision. Initial sTILs, CPS, CD68/CD163 findings, and mast-cell counts informed profile assignment; AR, the ratio, and morphology did not. CD68/CD163 values summarize each patient’s five-field mean, and mast-cell values summarize the mean across five fields selected by the hotspot protocol. Tryptase confirmed mast-cell identity. AR, androgen receptor; CPS, combined positive score; PD-L1, programmed death-ligand 1; sTILs, stromal tumor-infiltrating lymphocytes; TAM, tumor-associated macrophage; Q1–Q3, first to third quartile.

### 3.2. Tissue-Context Profiles

**Figure 1:**
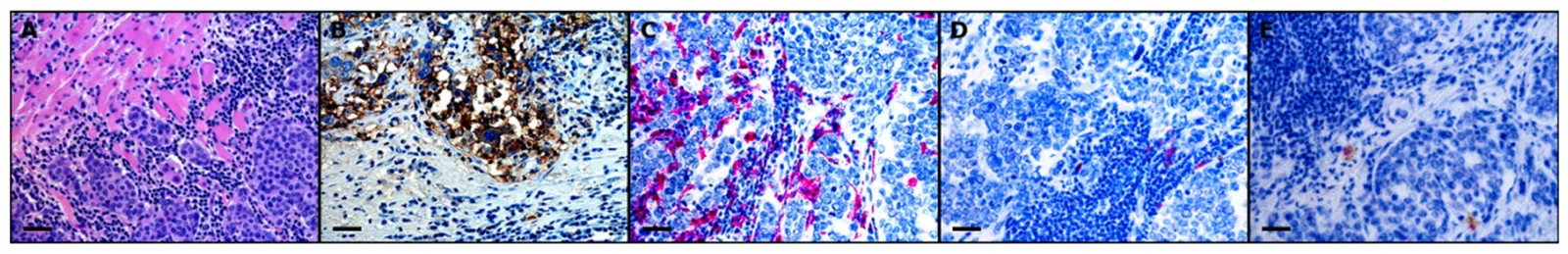
Representative histological and immunohistochemical features (**A**–**E**) from the same Profile I case. (**A**) Hematoxylin and eosin staining showing invasive breast carcinoma with conspicuous stromal lymphocytic infiltration. (**B**) Representative PD-L1 immunohistochemistry from a tumor with CPS ≥ 10. (**C**) CD68 immunohistochemistry highlights abundant CD68-positive TAMs, consistent with the CD68-high pattern. (**D**) CD163 immunohistochemistry shows sparse CD163-positive TAMs, consistent with the CD163-low pattern. (**E**) Dual CD117/tryptase immunohistochemistry demonstrates rare stromal mast cells showing same-cell CD117 positivity (red) and granular tryptase positivity (brown), consistent with the rare mast-cell hotspot category. Original magnification, ×200 for all panels; scale bars, 100 μm.

**Figure 2:**
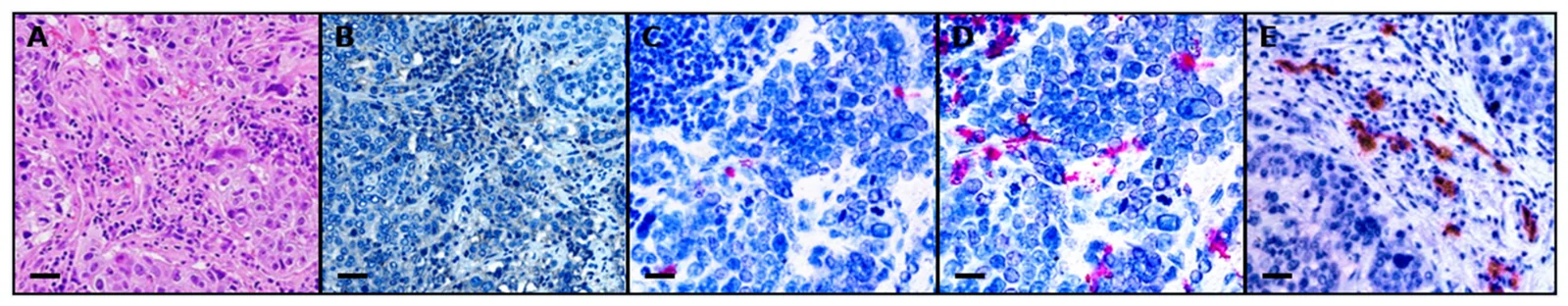
Representative histological and immunohistochemical features (**A**–**E**) from the same Profile II case. (**A**) Hematoxylin and eosin staining showing invasive breast carcinoma with relatively sparse stromal lymphocytic infiltration. (**B**) Representative PD-L1 immunohistochemistry from a tumor with CPS < 10. (**C**) CD68 immunohistochemistry shows relatively few CD68-positive TAMs, consistent with the CD68-low pattern. (**D**) CD163 immunohistochemistry highlights abundant CD163-positive TAMs, consistent with the CD163-high pattern. (**E**) Dual CD117/tryptase immunohistochemistry demonstrates numerous stromal mast cells showing same-cell CD117 positivity (red) and granular tryptase positivity (brown), consistent with the dense mast-cell hotspot category. Original magnification: ×200 (**A**,**B**) and ×400 (**C**–**E**). Scale bars: 100 μm (**A**,**B**) and 50 μm (**C**–**E**).

**Figure 3:**
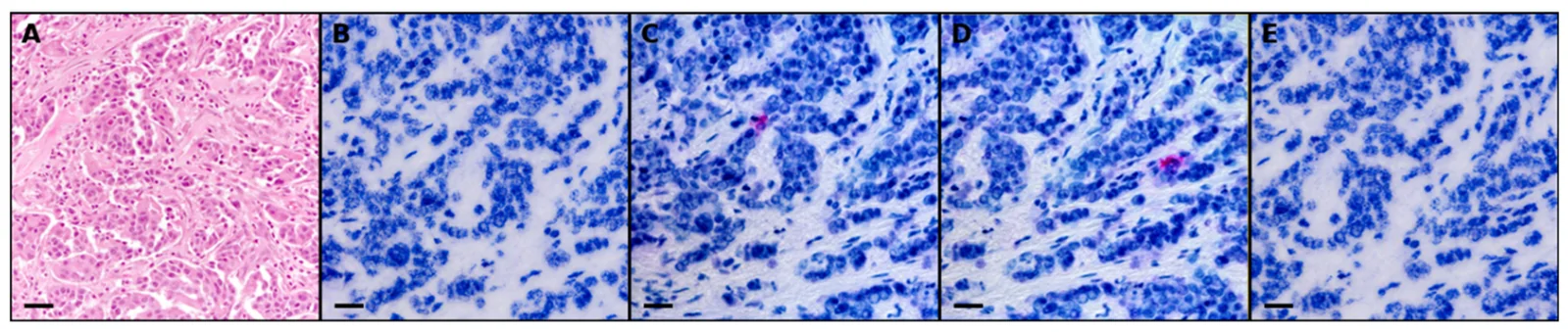
Representative histological and immunohistochemical features (**A**–**E**) from the same Profile III case. (**A**) Hematoxylin and eosin staining showing invasive breast carcinoma with minimal stromal lymphocytic infiltration. (**B**) Representative PD-L1 immunohistochemistry from a tumor with CPS < 10. (**C**) CD68 immunohistochemistry shows sparse CD68-positive TAMs, consistent with the CD68-low pattern. (**D**) CD163 immunohistochemistry similarly shows sparse CD163-positive TAMs, consistent with the CD163-low pattern. (**E**) Dual CD117/tryptase immunohistochemistry shows no stromal mast cells detected with same-cell CD117 (red) and tryptase (brown) coexpression, consistent with the not-detected mast-cell category. Original magnification: ×200 (**A**) and ×400 (**B**–**E**). Scale bars: 100 μm (**A**) and 50 μm (**B**–**E**).

Initial blinded review identified Profile I in seven tumors (29.2%), Profile II in 13 (54.2%), and Profile III in four (16.7%). Table 3 presents the initial sTIL findings, the numerical estimates from the second assessment, the original quantitative marker measurements, and accompanying morphology. Second-assessment sTIL estimates ranged from 19.1% to 40.0% in Profile I, from 1.0% to 9.2% in Profile II, and from 0.25% to 0.88% in Profile III, within the initial broad bands. These bands do not imply identical scores between assessments. Representative histological and immunohistochemical features are shown in Figure 1, Figure 2 and Figure 3.

Dual staining was technically adequate in all tumors. CD117-positive/tryptase-positive stromal mast cells were detected in 20 cases and not detected in four. No separate non-epithelial CD117-positive/tryptase-negative population was observed in the evaluated fields.

Profile I was characterized by sTIL levels of 15–40%, CPS ≥ 10, a CD68-high/CD163-low TAM pattern, and a rare mast-cell hotspot category. Androgen receptor was negative in all seven tumors, all of which were invasive breast carcinomas of no special type.

Profile II showed sTIL levels of 1–10%, CPS < 10, a CD68-low/CD163-high TAM pattern, and a dense mast-cell hotspot category. Androgen receptor expression was more heterogeneous, with positivity in six of 13 tumors; AR was not used for profile assignment. Twelve tumors were of no special type, and one showed apocrine differentiation.

Profile III showed minimal sTILs, CPS < 10, low CD68/CD163 counts, and no mast cells detected in the evaluated fields. All four tumors were AR-negative. Morphology included one carcinoma with pleomorphic features, one metaplastic carcinoma with chondroid differentiation, one with squamous differentiation, and one carcinoma of no special type. The CD163/CD68 ratio was within the study-defined balanced interval in all four cases but at low absolute counts.

### 3.3. Clinicopathological and Germline Comparisons

LVI was present in 4/7 Profile I, 1/13 Profile II, and 3/4 Profile III tumors. In the separate secondary two-test family, an exploratory between-profile difference was observed for LVI (exact *p* = 0.012; Benjamini–Hochberg-adjusted *p* = 0.023; Cramér’s V = 0.604). PNI was present in 3/7, 1/13, and 1/4 tumors, respectively, without evidence of a between-profile difference (exact *p* = 0.190; adjusted *p* = 0.190; Cramér’s V = 0.380).

All four germline P/LP-positive patients belonged to Profile I: 4/7 (57.1%; exact 95% CI, 18.4–90.1%), versus 0/13 (0%; 95% CI, 0–24.7%) and 0/4 (0%; 95% CI, 0–60.2%) in Profiles II and III, respectively. The global exact *p* value was 0.006 (Benjamini–Hochberg-adjusted *p* = 0.030). Omitting any one of the four positive patients gave *p* = 0.014 and adjusted *p* = 0.068. Age, Ki-67, cN status, and clinical stage showed no clear between-profile differences; estimates and effect sizes are reported in Table 4.

### 3.4. Quantitative Assessment of the Tissue Profiles

Using the second-assessment sTIL percentages and the four original quantitative measurements, Ward clustering favored three clusters (mean silhouette, 0.631) over two, four, or five clusters (0.452, 0.538, and 0.494, respectively). The 7/13/4 partition matched the initial pathology profiles exactly (ARI = 1.000). The same assignments were obtained after rounding sTILs to five-percentage-point increments (silhouette, 0.609) and after excluding sTILs (0.617). These results describe consistency within the 24-case dataset, not reproducibility in an independent cohort.

### 3.5. Anthropometric, Treatment Response, and Follow-Up Analyses

Median BMI was 23.1, 25.7, and 29.0 kg/m^2^ in Profiles I–III, respectively; obesity occurred in 0/7, 3/13, and 2/4 patients, respectively (Table 4). Neither exploratory comparison showed a clear difference. Among the 16 neoadjuvant-treated patients, all with evaluable final surgical pathology, pCR occurred in 3/4, 3/9, and 1/3 cases, respectively (exact *p* = 0.405; Table 5). Primary treatment was completed in 23 patients; one Profile II patient had not completed it at last contact. No progression or recurrence before treatment completion was recorded. Five posttreatment recurrences were documented: one in Profile I, three in Profile II, and one in Profile III. These represent 5/24 (20.8%) of the full cohort and 5/23 (21.7%) of patients who completed primary treatment. Among these five patients, the median time from biopsy to first recurrence was 18 months (Q1–Q3, 12–21; range, 11–24). First recurrence was locoregional only in one patient, distant only in three, and both locoregional and distant in one. Seven deaths occurred: one, three, and three in Profiles I–III, respectively. Median follow-up was 35 months (observed range, 5–60); the exploratory OS comparison did not establish a between-profile difference (log-rank *p* = 0.237).

## 4. Discussion

In this series of pretreatment TNBC core biopsies, morphological and immunohistochemical review revealed recurring combinations of lymphocytic infiltration, PD-L1 expression, macrophage counts, and mast-cell abundance, from which three tissue profiles were recognized. Profile I combined higher sTILs and PD-L1 CPS with a CD68-high/CD163-low pattern and rare mast cells. Profile II had lower sTILs and CPS, a CD68-low/CD163-high pattern, and dense mast-cell hotspots. Profile III had minimal sTILs, low CD68/CD163 counts, and no mast cells detected in the evaluated fields. The M1/M2 model has long been used to describe macrophage polarization, but TAMs in human tumors span a continuum of phenotypic and functional states that cannot be reliably assigned using a single immunohistochemical marker [19]. CD68 is a broad macrophage marker, whereas CD163 is associated with an immunoregulatory phenotype; neither marker alone establishes functional polarization [19,20,21]. Accordingly, these profiles describe macrophage and mast-cell composition alongside the degree of lymphocytic infiltration.

The higher sTIL levels and PD-L1 CPS in Profile I are consistent with the relationship between lymphocytic infiltration and checkpoint expression described in TNBC [14,15,16,17,18]. They identify a lymphocyte-richer tissue pattern within this cohort. Their clinical interpretation nevertheless depends on disease setting: CPS ≥ 10 is used for treatment selection in advanced TNBC [17,36], whereas KEYNOTE-522 enrolled patients with stage II–III disease irrespective of PD-L1 status [37]. The CPS categories used here therefore describe the biopsy findings and should not be interpreted as immunotherapy eligibility criteria for these nonmetastatic patients. Comparisons of PD-L1 findings require attention to the assay, scoring system, and tumor setting [17,39,40,41].

The macrophage findings added a second distinction between the profiles. CD68-positive counts were higher in Profile I, whereas CD163-positive counts were higher in Profile II; both were low in Profile III. These observations complement previous studies examining macrophage markers alongside lymphocytic infiltration in TNBC [20,21]. They describe differences in immunophenotypic composition, rather than M1 or M2 functional polarization [19].

For clinicians and pathologists, this approach may help organize the findings in pretreatment biopsies and select contrasting cases for molecular or spatial studies. Integrated assessment of morphology and the local microenvironment has also been explored in other tumor settings [42]. Here, it raises questions about epithelial differentiation, hereditary predisposition, and treatment response. This is a research use, not a basis for selecting germline tests or systemic treatment. Clinical application would require reproducible assignment criteria and evidence of added value beyond established clinicopathological variables.

Same-cell CD117/tryptase staining confirmed the mast cells included in the counts. Their distribution provided another distinction between the profiles, with denser hotspots in Profile II, rare cells in Profile I, and no cells detected in the evaluated Profile III fields. Studies linking mast cells with angiogenic features in breast cancer provide a rationale for examining this component alongside lymphocytes and macrophages [22,23]. Our findings identify a difference in local mast-cell counts; whether it has functional significance remains to be established.

All four germline *BRCA1*/*BRCA2* P/LP-positive patients belonged to Profile I. This was the most notable germline finding, but its precision was limited: the P/LP proportion in Profile I was 4/7 (57.1%; 95% CI, 18.4–90.1%). The adjusted *p* value increased from 0.030 to 0.068 when any one of the four positive cases was omitted. Three patients carried *BRCA1* variants (two truncating and one canonical splice-site variant), and one carried a *BRCA2* frameshift variant. Although these clinically classified P/LP variants are compatible with loss of function, their effects on the individual tumors were not tested.

Broader germline studies have documented the genetic and clinicopathological heterogeneity of TNBC [29]. A study comparing *BRCA1*/*BRCA2*-mutated and non-mutated TNBC found broadly similar immune organization, despite more frequent TIL-positive tumors in the mutated group [43]. Our observation therefore raises a question for further study rather than establishing a *BRCA*-associated immune phenotype. The selected testing population is important when interpreting it: patients referred because of age or personal and family cancer history may differ from those who were not tested.

Clustering using the second sTIL assessment and the original marker measurements reproduced the initial pathology assignments. Agreement was retained when the sTIL estimates were rounded or omitted. This addresses the consistency of the measured features within this series but does not show that all five markers are necessary or that they provide better clinical discrimination than a single marker. The initial sTIL bands and mast-cell categories already separated the profiles. Since the analysis used the same tumors and overlapping features, it remains an internal comparison.

BMI and obesity did not show clear differences across profiles. Among patients receiving neoadjuvant chemotherapy, pCR occurred in 3/4 Profile I, 3/9 Profile II, and 1/3 Profile III cases. Although the proportion was numerically higher in Profile I, the small denominators and wide confidence intervals prevent a reliable comparison. All 16 patients received an anthracycline- and taxane-containing backbone, but this alone does not exclude differences in other treatment or patient characteristics. Five posttreatment recurrences and seven deaths were documented, and the exploratory analyses did not establish prognostic differences between profiles. Larger cohorts with standardized treatment and follow-up are needed to assess clinical relevance.

Biopsy-detected LVI was present in 4/7, 1/13, and 3/4 tumors in Profiles I–III, respectively (adjusted *p* = 0.023). This secondary finding warrants further investigation, but interpretation is limited by the small numbers and the amount of tissue sampled. PNI was identified in only five tumors, without a clear between-profile difference. Tumor–nerve interactions are increasingly studied as part of the local tumor environment [44]; PNI on a core biopsy captures only one morphological aspect of these interactions.

This study has several limitations. It is a small, single-institution series with one pretreatment core biopsy per patient, unequal profile sizes, and only four germline P/LP-positive cases. Thirty-one of 74 institutional patients completed germline testing, and 24 met the tissue criteria. Detailed family history and ancestry data were unavailable for adjustment, so we could not assess how these factors influenced inclusion. The observed P/LP frequency and profile distribution should not be generalized to all patients with TNBC. Three of the four Profile III tumors had special or divergent morphology, which may also have contributed to their tissue findings.

Because the study describes profiles recognized on slide review, their marker distributions characterize the observed patterns rather than independently validate them. Interobserver reproducibility could not be assessed because the pathologists worked jointly without independent reader scores. The second sTIL reading used the same slides and readers; it was not an independent validation, and the incomplete retention of the original numerical scores prevented a test–retest comparison. The numerical categories remain exploratory, and CD163/CD68 ratios require caution at low absolute counts. Mast-cell counts describe five selected hotspots rather than whole-tumor density; raw cell counts may also vary with the stromal area within each field. LVI and PNI may be missed in a limited core biopsy.

The panel provided a limited view of immune composition. It did not distinguish lymphocyte subsets or their functional states, and CPS alone cannot establish an immune-inflamed phenotype. CD68/CD163 staining does not establish macrophage polarization, and CD117/tryptase coexpression does not establish mast-cell activation or function. Broader immune and spatial analyses are needed to investigate these questions and additional immunotherapy targets [45]. Tumor-level loss of heterozygosity, biallelic *BRCA* inactivation, homologous-recombination deficiency, and variant-specific functional effects were not assessed. Comparable results for other susceptibility genes were also unavailable across the cohort.

Finally, the small sample and event counts precluded reliable multivariable, gene-specific, or prognostic analyses. Follow-up was based on each patient’s last documented contact rather than a uniform observation period, and treatment modifications and completion were not modeled. Absence of a statistically significant outcome difference does not establish equivalence. Independent multicenter studies should evaluate explicit assignment criteria, observer agreement, and any added prognostic or treatment-related value before these profiles are used clinically.

## 5. Conclusions

In this selected cohort of 24 patients with TNBC, pretreatment biopsies showed three profiles with different combinations of lymphocytic infiltration, PD-L1 expression, CD68/CD163-positive macrophages, and mast-cell hotspots. Exploratory clustering reproduced the pathology-based assignments, supporting consistency within this series rather than independent validation. All four germline *BRCA1*/*BRCA2* P/LP-positive patients were in Profile I, an exploratory observation sensitive to the small number of positive cases and clinical selection for testing. Larger independent multicenter cohorts are required to establish reproducibility and clinical or prognostic value before these profiles can guide patient care.

## Figures and Tables

**Table 4:** Biopsy, clinical, germline, and anthropometric comparisons across the three tissue-context profiles.

Variable	Profile I (*n* = 7)	Profile II (*n* = 13)	Profile III (*n* = 4)	Unadjusted *p*	BH-Adjusted *p*	Effect Size
LVI present	4/7 (57.1%; 95% CI, 18.4–90.1%)	1/13 (7.7%; 95% CI, 0.2–36.0%)	3/4 (75.0%; 95% CI, 19.4–99.4%)	0.012	0.023	Cramér’s V = 0.604
PNI present	3/7 (42.9%; 95% CI, 9.9–81.6%)	1/13 (7.7%; 95% CI, 0.2–36.0%)	1/4 (25.0%; 95% CI, 0.6–80.6%)	0.190	0.190	Cramér’s V = 0.380
Documented germline *BRCA1*/*BRCA2* P/LP variant	4/7 (57.1%; 95% CI, 18.4–90.1%)	0/13 (0%; 95% CI, 0–24.7%)	0/4 (0%; 95% CI, 0–60.2%)	0.006	0.030	Cramér’s V = 0.697
Ki-67, median (Q1–Q3), %	70 (60–80)	60 (40–70)	50 (37.5–62.5)	0.258	0.645	η^2^_H_ = 0.037
Pretreatment cN-positive disease	4/7 (57.1%; 95% CI, 18.4–90.1%)	6/13 (46.2%; 95% CI, 19.2–74.9%)	3/4 (75.0%; 95% CI, 19.4–99.4%)	0.747	0.856	Cramér’s V = 0.210
Age, median (Q1–Q3), years	56 (52–62)	66 (45–72)	62.5 (58–63.3)	0.837	0.856	η^2^_H_ = 0.000
Pretreatment clinical stage III	4/7 (57.1%; 95% CI, 18.4–90.1%)	5/13 (38.5%; 95% CI, 13.9–68.4%)	2/4 (50.0%; 95% CI, 6.8–93.2%)	0.856	0.856	Cramér’s V = 0.167
BMI, median (Q1–Q3), kg/m^2^	23.1 (22.6–26.2)	25.7 (21.6–28.6)	29.0 (24.5–32.1)	0.715	—	η^2^_H_ = 0.000
Obesity, BMI ≥30 kg/m^2^	0/7 (0%)	3/13 (23.1%)	2/4 (50.0%)	0.147	—	Cramér’s V = 0.405

Exact 95% confidence intervals are Clopper–Pearson intervals. The Benjamini–Hochberg adjustment was applied separately to the five main comparisons (germline status, Ki-67, cN, age, and stage) and to the two secondary biopsy comparisons (LVI and PNI). BMI and obesity were additional exploratory comparisons with unadjusted *p* values, indicated by a dash in the adjusted-p column. Categorical variables used exact tests; age and Ki-67 used permutation Kruskal–Wallis tests and BMI the Kruskal–Wallis test. BH, Benjamini–Hochberg; BMI, body mass index; CI, confidence interval; cN, clinical N category; LVI, lymphovascular invasion; P/LP, pathogenic or likely pathogenic; PNI, perineural invasion; η^2^_H_, eta-squared based on the Kruskal–Wallis statistic; Q1–Q3, first to third quartile.

**Table 5:** Exploratory treatment response and follow-up outcome comparisons across the three tissue-context profiles.

Variable	Profile I (*n* = 7)	Profile II (*n* = 13)	Profile III (*n* = 4)	Exploratory Comparison
Neoadjuvant therapy	4/7 (57.1%)	9/13 (69.2%)	3/4 (75.0%)	Descriptive
pCR among neoadjuvant-treated patients	3/4 (75.0%; 95% CI, 19.4–99.4%)	3/9 (33.3%; 95% CI, 7.5–70.1%)	1/3 (33.3%; 95% CI, 0.8–90.6%)	Exact *p* = 0.405; Cramér’s V = 0.364
OS events (deaths)	1/7 (14.3%; 95% CI, 0.4–57.9%)	3/13 (23.1%; 95% CI, 5.0–53.8%)	3/4 (75.0%; 95% CI, 19.4–99.4%)	OS: global log-rank *p* = 0.237
Posttreatment recurrences	1/7 (14.3%; 95% CI, 0.4–57.9%)	3/13 (23.1%; 95% CI, 5.0–53.8%)	1/4 (25.0%; 95% CI, 0.6–80.6%)	Descriptive

Values are *n*/*N* (%) unless otherwise stated; exact 95% confidence intervals are Clopper–Pearson intervals. pCR was defined as ypT0/is ypN0 and was evaluable in all 16 neoadjuvant-treated patients, who received an anthracycline- and taxane-containing chemotherapy backbone. The pCR and OS comparisons were exploratory, with nominal *p* values unadjusted for multiple testing. Death and recurrence percentages use the full profile denominators and describe observed events, not cumulative incidence. The log-rank *p* value refers to the time-to-event comparison of OS across all three profiles. Primary treatment was completed in 7, 12, and 4 patients in Profiles I–III; the remaining Profile II patient had not entered posttreatment observation. The conditional recurrence proportion among those completing treatment was 5/23 (21.7%). Median follow-up by reverse Kaplan–Meier was 35 months (observed range, 5–60). CI, confidence interval; OS, overall survival; pCR, pathologic complete response.

## Data Availability

De-identified analytical data supporting the reported results are available from the corresponding author upon reasonable request and subject to institutional, ethical, and data-protection requirements applicable to coded clinical and germline information. The R scripts used for the clustering, sensitivity, and exploratory outcome analyses are available from the corresponding author upon reasonable request.
